# Editorial: Multi-omics profiling of unique niches to reveal the microbial and metabolite composition

**DOI:** 10.3389/fmicb.2022.997191

**Published:** 2022-08-19

**Authors:** Roshan Kumar, Vasvi Chaudhry, Om Prakash

**Affiliations:** ^1^Post-Graduate Department of Zoology, Magadh University, Bodh Gaya, India; ^2^Department of Microbial Interactions, IMIT/ZMBP, University of Tübingen, Tübingen, Germany; ^3^National Centre for Microbial Resource (NCMR), National Centre for Cell Science, Pune, Maharashtra, India

**Keywords:** multi-omics, microbial composition and distribution, niches, proteomics, metagenomics, transcriptomics, metabolomics

It is an exciting time in the field of Microbiology. With the rise of the era of OMICS, we have witnessed an explosion of modern multi-omics technologies. These include (meta)genomics, transcriptomics, proteomics, meta-metabolomics, and culturomics in different aspects of basic and applied research. The use of OMICS has greatly enhanced our understanding of the structure, structure-related functions, and interactions among microbes with biotic and abiotic components of ecosystems and revealed their role in offering different ecosystem services (Kumar et al., 2022). Considering the extensive use and versatility of OMICS techniques in different disciplines of life science, we created this Research Topic “*Multi-omics profiling of unique niches to reveal the microbial and metabolite composition*” to understand the role of microbes in their respective niches in the presence and absence of certain variables. In this special issue, we selected and published 11 articles contributed by 76 authors. The articles were selected based on their significance in understanding the current state of research on the topic. We take the opportunity here to reflect the significant contributions of these articles.

The first article in this issue demonstrates the impact of dietary supplements, namely quercetin and rice bran, on enriching beneficial gut microbes in a mini-bioreactor array system, i.e., an *in vitro* model system without the confounding host factors (Ghimire et al.). According to the study's findings, rice bran induces substantial changes in gut- microbial diversity when compared with quercetin. In contrast to this, in quercetin supplementation, the *Acidaminococcus intestine* was significantly enriched along with increased iso-butyrate production. Another study in this issue utilizes phosphate-solubilizing bacteria (PSB) isolated from the sweet potato rhizosphere. Multi-omics analysis of these strains demonstrated the upregulation of genes involved in the gluconic acid synthesis and the tricarboxylic acid cycle (Ding et al.). The study concludes that increased gluconic and malic acid, due to up-regulation of genes, is responsible for enhanced phosphate-solubilizing potential.

Further, one study with *Klebsiella pneumoniae* x546, analyzes the production of 1, 3-propanediol (1, 3-PDO) and reports improved production with betaine and shorter fermentation time under controlled pH conditions (Wang et al.). This study used the proteomics approach to identify proteins expressed during fermentation, such as homoserine kinase (ThrB), and in 1, 3-PDO biosynthesis, such as lactate dehydrogenase (LDH), DhaD, DhaK, and BudC.

In another study, the authors reveal the relationship between antibiotic sensitivity and lipid metabolism using a marine bacterium *Meyerozyma guilliermondii* GXDK6 (Sun et al.). They demonstrated that under 10% NaCl stress, expression of AYR1 and NADPH-dependent 1-acyl-dihydroxyacetone phosphate reductase was inhibited, which triggered the intracellular accumulation of glycerol and weakened the budding and proliferation process of cells. They also reported that NaCl stress enhanced the organism's sensitivity to fluconazole by inhibiting the expression of drug target proteins lanosterol 14-alpha demethylase.

Another article examines whether a digital therapeutic intervention tailored to genomic SNPs and gut-microbiome signals could reduce symptoms of functional gastrointestinal disorders (FGIDs) using the datasets from individuals who have successfully lost weight (Kumbhare et al.). A logistic regression model was trained to distinguish FGID status among subjects enrolled in digital therapeutics care programs based on demographic, genetic, and baseline microbiome data.

In their paper, Candeliere et al. assembles, bins, and mines the intestinal metagenomes of 60 healthy individuals. This study evaluated the profiles of β-glucuronidases (GUS), an important enzyme that plays a pivotal role in metabolizing drugs in the gastrointestinal (GI) tract (Pollet et al., 2017) among healthy individuals from five geographically diverse cohorts (Candeliere et al.). Only the Ethiopia cohort (ETH) had significantly fewer GUS-encoding bacteria when compared to the other cohorts. The bacterial species contributing to different GUS categories belonged to genus *Bacteroides, Faecalibacterium*, and *Eubacterium*.

An additional metagenomic analysis published in this issue found abundant sulfur-metabolizing genetic repertoires and microbes with established S-cycling capabilities, including *Pseudomonas, Thioalkalivibrio, Desulfovibrio*, and *Desulfobulbaceae* (Nagar et al.). As evident from the diversity of sulfur-oxidizing bacteria (SOB) and sulfate-reducing bacteria (SRB) with conserved (r)dsrAB, it is likely to be a necessary adaptation for microbial fitness at this site. To get a detailed insight into the metal fungus interactions and to develop the technology of Mycoremediation of heavy metal contaminated sites, another paper in the issue examines the protein expression in *Aspergillus fumigatus* PD-18 in response to heavy metals stress (5 mg/L of Cr, Cd2+, Cu2+, Ni2+, Pb2+, and Zn2+) using the proteomics approach (Dey et al.). Signaling and cellular processing were the most prevalent functional classes under these conditions. Using protein-protein interaction network analysis, authors report that cytochrome-c oxidase and 60S ribosomal protein had crucial roles in detoxifying multi-metals.

A review article explores and compares the effect of metabolic and microbiota alterations on disease progression in patients with COVID-19 and inflammatory bowel disease (Cortes et al.). The study emphasizes that both the conditions are associated with decreased Firmicutes populations, with a specific reduction in *Faecalibacterium prausnitzii* cells, which produces different kinds of short-chain fatty acid (SCFA) under anaerobic conditions. The study also examines how gut dysbiosis, inflammation, oxidative stress, and energy demand affect key metabolites such as tryptophan, phenylalanine, histidine, glutamine, succinate, and citrate levels in the gut.

Using a comparative genomic approach, Mohapatra et al. investigates the presence of three genomic islands, ICEnahCSV86, PBGI-1, and PBGI-2 (GIs, > 50 Kb size), in an aromatic-compound degrading soil bacterium, *Pseudomonas bharatica* CSV86T. Additionally, they elucidate the role of MGEs in the adaptation, niche colonization, and species competitive behavior of strain CSV86T in contaminated environments.

In one metagenomic study by Vasudeva et al., the authors analyze the samples collected from activated sludge of an industrial effluent treatment plant. It highlights the abundance of genes involved in xenobiotic degradation pathways and antimicrobial resistance. In both samples, glycosyl hydrolases and glycosal transferases were the most abundant carbohydrate-active enzyme classes compared to other polysaccharide enzyme classes.

In conclusion, the special issue featured several fascinating articles illustrating the importance of genomics, metagenomics, transcriptomics, proteomics, and metabolomics to understand different niches' microbes and microbial processes. The editors believe that this issue has successfully achieved reporting recent investigations on microbial processes relevant to a variety of microbial processes in a variety of environmental niches.

## Author contributions

All authors listed have made a substantial, direct, and intellectual contribution to the work and approved it for publication.

## Conflict of interest

The authors declare that the research was conducted in the absence of any commercial or financial relationships that could be construed as a potential conflict of interest.

## Publisher's note

All claims expressed in this article are solely those of the authors and do not necessarily represent those of their affiliated organizations, or those of the publisher, the editors and the reviewers. Any product that may be evaluated in this article, or claim that may be made by its manufacturer, is not guaranteed or endorsed by the publisher.
